# Moran's I-driven habitat radiomics: A biologically plausible and temporally robust approach for risk stratification of lung adenocarcinoma invasiveness

**DOI:** 10.1016/j.ejro.2026.100792

**Published:** 2026-07-14

**Authors:** Lingqi Gao, Sifan Chen, Bo Li, Maolu Tan, Xiaogang Chen, Fajin Lv

**Affiliations:** a Department of Radiology, The First Affiliated Hospital of Chongqing Medical University, Chongqing 400016, China; b Department of Radiology, Renji Hospital Affiliated to Shanghai Jiao Tong University School of Medicine, Shanghai 200127, China; c Department of Radiology, The Tongnan District People's Hospital, Chongqing, China

**Keywords:** Pulmonary nodules, Invasiveness, Habitat radiomics, Moran’s I, Interpretability

## Abstract

**Background:**

Preoperative differentiation of pulmonary nodules into preinvasive (AAH/AIS), minimally invasive (MIA) and invasive adenocarcinoma (IAC) subtypes is vital for clinical decision-making, but conventional radiomics lacks biological interpretability and reproducibility. This study proposed a Moran’s I-driven habitat radiomics approach to evaluate the temporal stability of invasiveness risk assessment on serial CT.

**Methods:**

A total of 614 patients from two centers were enrolled, including a training set (n = 400), an internal validation set (n = 104), and an independent external testing set (n = 110). Notably, the external set comprised patients with serial longitudinal CT scans (preoperative, 3-, 6-, and 12-month) to validate temporal generalizability. Local Moran’s I partitioned tumors into four habitats by spatial autocorrelation. Feature reproducibility was verified via image perturbation, and an optimal combined model was built with robust habitat features and compared with conventional radiomics. SHapley Additive exPlanation (SHAP) analysis was employed to revealed associations between habitat features and pathological cell density, offering hypothesis-generating biological insights.

**Results:**

The Combined model demonstrated superior discrimination, achieving a macro-averaged AUC of 0.830 in the validation set and 0.854 in the external testing set. Specifically, the AUCs were 0.845 for AAH/AIS, 0.787 for MIA, and 0.931 for IAC. In the temporal robustness analysis, the Combined model outperformed conventional radiomics across all preoperative follow-up time points, yielding more reliable sensitivity for invasive lesions. SHAP analysis revealed that habitat features correlated with pathological cell density, offering intelligible biological insights.

**Conclusions:**

This biologically plausible and temporally stable model enables noninvasive risk stratification of lung adenocarcinoma invasiveness and may support longitudinal surveillance of pulmonary nodules.

## 1 Introduction

The increasing detection of lung adenocarcinoma (LUAD), a leading cause of cancer mortality, is closely linked to the widespread adoption of low-dose computed tomography (LDCT) screening [1], [2]. The 2021 World Health Organization classification now recognizes atypical adenomatous hyperplasia (AAH) and adenocarcinoma in situ (AIS) as glandular precursor lesions [3]. Critically, minimally invasive adenocarcinoma (MIA) and invasive adenocarcinoma (IAC) require different surgical management strategies [4]. MIA and preinvasive lesions (AAH/AIS) are favorably managed with sublobar resection, offering a nearly 100% 5-year survival rate [5]. In contrast, IAC carries a poorer prognosis and typically necessitates lobectomy combined with systematic lymph node dissection [6]. However, distinguishing these subtypes on preoperative CT remains challenging due to overlapping radiological features, highlighting the need for precise non-invasive biomarkers [7], [8].

Radiomics addresses this challenge by extracting numerous high-dimensional quantitative features from medical images, thereby quantifying subtle, visually imperceptible details into computable data [9]. Previous studies have established its role as a reliable tool for predicting the invasiveness of lung cancer [10], [11]. However, the performance of radiomics models suffers from poor robustness and considerable inter-study variability, largely attributable to the poor repeatability and reproducibility of features across different imaging platforms and protocols [12]. Besides, conventional radiomics lacks biological interpretability [13]. The extracted features are data-driven mathematical descriptors without direct pathological correlates, undermining clinical confidence [14]. Moreover, conventional radiomics treats the tumor as a homogeneous entity, extracting averaged features that mask critical intratumoral heterogeneity (ITH), a hallmark of cancer aggressiveness. To address this, habitat analysis decomposes the tumor into subregions with distinct phenotypes. Yet, current habitat approaches predominately rely on unsupervised clustering algorithms, which are inherently data-driven and stochastic. These methods often lack physiological interpretability and suffer from poor reproducibility across different scanners, limiting their clinical trust and translation.

To overcome these limitations, we propose a novel, biologically plausible habitat paradigm using Local Moran's I. Unlike initialization-sensitive clustering algorithms such as K-means and Gaussian Mixture Models (GMM), Moran's I quantifies spatial autocorrelation to measure how voxel intensities cluster locally. This attribute-spatial association can be leveraged to robustly partition tumors into habitats. These habitats may reflect underlying pathological architecture (e.g., high-density core vs. ground-glass margin). The interpretations are biologically plausible.

Consequently, this study aims to develop an explainable habitat radiomics model to discriminate AAH/AIS, MIA, and IAC. Distinct from previous works, we emphasized two critical translational aspects: Rigorous robustness validation: assessing feature and clustering stability against image perturbations to ensure reproducibility; and temporal robustness: evaluating the model's predictive performance serial preoperative CT scans, thereby validating its temporal generalizability for active surveillance of pulmonary nodules.

## 2 Methods

### 2.1 Patients’ characteristics

**Fig. 1:**
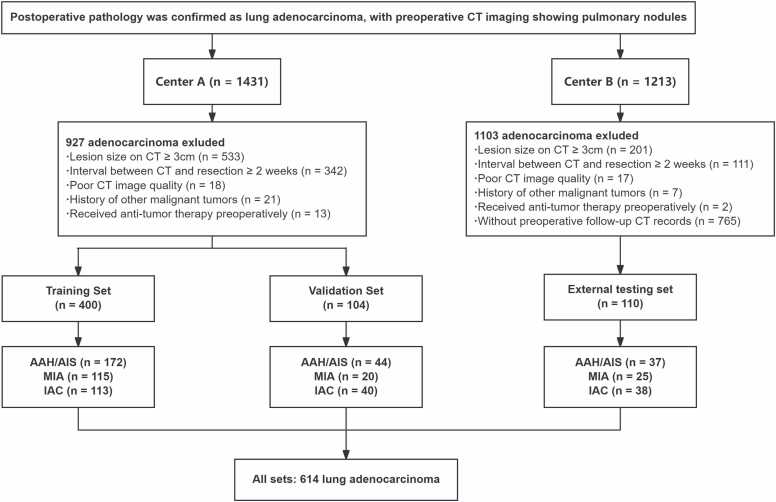
Recruitment process of LUAD patients.

This retrospective study enrolled 1431 patients with surgically resected LUAD from Center A (Jan 2018 to Jul 2023). The study protocol was approved by the institutional ethics committee (IRB, No. 2024-046-01), and the requirement for informed consent was waived due to the use of anonymized data and the retrospective nature of the study. From Center A (Jan 2018–Jul 2023), 504 eligible patients were included based on the following criteria: [1] preoperative chest CT slice thickness ≤ 1.5 mm; [2] pathological confirmation of primary LUAD. Exclusion criteria included: [1] lesion size on CT ≥ 3 cm; [2] interval between CT and resection ≥ 2 weeks; and [3] poor CT image quality; [4] with other types of malignant tumors; [5] preoperative anti-tumor therapy. These patients were randomly partitioned into a training set (n = 400) and an internal validation set (n = 104) with stratification (approximately 8:2 split). To evaluate temporal generalizability, we included an independent cohort from Center B (Jan 2015–Dec 2023). A total of 110 patients who underwent at least three serial preoperative CT scans (intervals: <2 weeks, 3–6 months, and 6–12 months pre-surgery) were included using the same criteria. A flowchart of patient selection is provided in Fig. 1.

### 2.2 Image acquisition and image segmentation

**Fig. 2:**
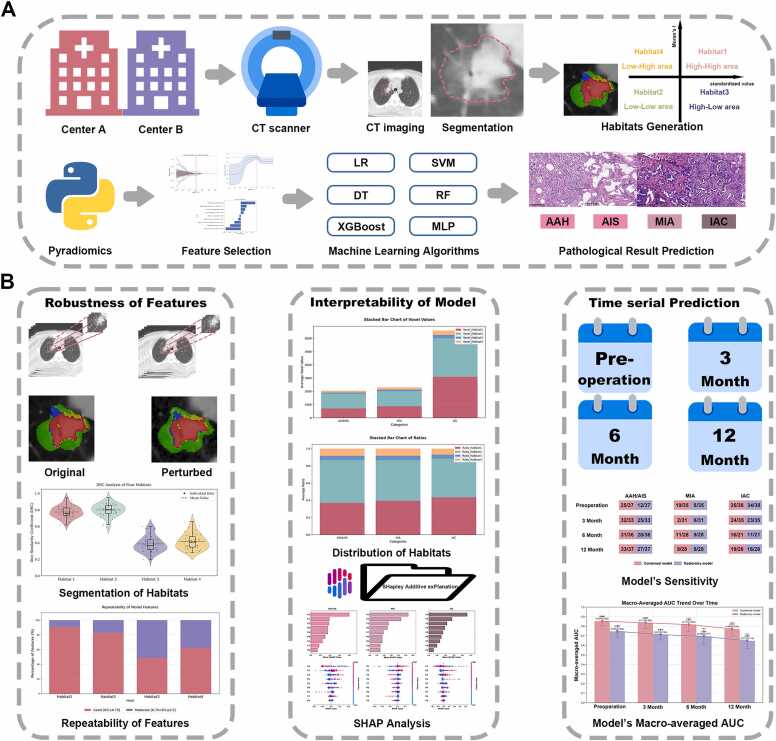
Overview of our workflow. LR, Logistic Regression; SVM, Support Vector Machine; XGBoost, Extreme Gradient Boosting; RF, Random Forest; DT, Decision Tree; MLP, Multilayer Perceptron; AAH, Atypical Adenomatous Hyperplasia; AIS, Adenocarcinoma in Situ; MIA, Minimally Invasive Adenocarcinoma; IAC, Invasive Adenocarcinoma.

The patients underwent unenhanced spiral CT scans from the supraclavicular fossa to the lung bases. CT scans were performed using various CT scanners. Detailed CT scan and reconstruction parameters are provided in Table S2. Two radiologists, in a blinded manner, employed ITK-SNAP, version 4.0.1 (https://www.itksnap.org/) to manually segment the regions of interest (ROIs) of tumors. Each tumor was initially segmented by a radiologist with over 3 years of work experience and subsequently reviewed by another radiologist with over 8 years of experience. In case of discrepancies, a senior radiologist with more than 30 years of experience would either perform revisions to the segmentation results or conduct a new segmentation. A comprehensive overview of our workflow is presented in Fig. 2.

### 2.3 Image assessment and standardization

Comprehensive radiomics workflow was established following best-practice guidelines, with study quality evaluated using the Radiomics Quality Score [9] (RQS; Supplementary Appendix 1). The detailed component scores are provided in Supplementary Table S6. To mitigate intervendor and intercenter variations, original images underwent multistep standardization based on recommendations from the Imaging Biomarker Standardization Initiative. Specifically, images were resampled to 1 × 1 × 1 mm³ resolution via linear interpolation, followed by voxel value discretization with a bin width of 25 HU. Finally, both habitat and radiomics features were harmonized using the ComBat method [15] (https://github.com/Jfortin1/ComBatHarmonization). Each distinct CT scanner model, regardless of its center, was treated as an independent batch to correct for both inter-center and intra-center scanner variations.

### 2.4 Moran’s I-based habitat clustering

CT images and their corresponding mask files were first inputted into the analysis pipeline. Spatial attributes of the target regions were systematically analyzed, followed by the extraction of voxel sets corresponding to each ROI. A neighborhood weight matrix was constructed using Euclidean distance as the core metric, and the Local Moran’s I for each individual voxel was further computed to quantify the spatial correlation patterns among adjacent voxels. Of note, the habitat analysis was performed independently for each lesion rather than jointly across the cohort. For every tumor, we calculated Local Moran's I using its own voxel set and stratified its voxels into four habitats based on local intensity-neighborhood relationships. Subsequently, based on the relationship between the intensity of a target voxel and the mean intensity of its local neighborhood, all voxels were stratified into four distinct habitats:

Habitat 1 (High-High): High-intensity voxels surrounded by high-intensity neighbors (indicating solid aggregation).

Habitat 2 (Low-Low): Low-intensity voxels surrounded by low-intensity neighbors.

Habitat 3 (High-Low) & Habitat 4 (Low-High): Voxels with intensities diverging from their neighbors (indicating transition zones).

This categorization was performed using criteria derived from the magnitude and direction of the Local Moran's index (Supplementary Appendix 2).

### 2.5 Image perturbation and feature extraction

To simulate a retest scenario for assessing the repeatability of radiomics features, image perturbations were applied using the Medical Image Radiomics Processor Python toolkit [16], version 1.2.0 (https://github.com/oncoray/mirp). Specifically, we applied a combination of rotational, translational, and Gaussian noise perturbations, consistent with established methodologies reported in previous literature [17], [18]. Gaussian noise was introduced with a mean of zero and a standard deviation proportional to the image intensity variability. Translation was applied by shifting the voxel grid by a fraction of the voxel spacing along each axis, while in-plane rotation was performed around the z-axis with an angle of 0.5°. The detailed parameter settings for each perturbation type are provided in Supplementary Table S7. All perturbations were implemented using standardized functions from the Medical Image Radiomics Processor package to ensure reproducibility and methodological consistency across the dataset. Following perturbation, both original and perturbed images underwent habitat clustering and subsequent radiomics feature extraction from habitat subregions. Using PyRadiomics [19], version 3.0.1 (https://github.com/AIM-Harvard/pyradiomics/), a total of 107 non-filtered radiomics features were extracted. Detailed characteristics are summarized in Table S3.

### 2.6 Repeatability analysis for precise radiomics features

The stability of clustered habitats, assessed by the Dice Similarity Coefficient (DSC) between each original and perturbed image pair, was evaluated to quantify habitat similarity. DSC was caculated as follows.

(1) $$\text{DSC }=\;\frac{2\text{|A}\cap \text{B|}}{\text{|A| }+\text{ |B|}}$$

The intraclass correlation coefficient (ICC) was employed to measure the robustness of radiomics features, calculated using a one-way random-effects model under the absolute agreement definition with a single-rater/measurement assumption. An ICC value of 1 indicates perfect robustness, while 0 suggests complete unreliability. Features derived from each habitat subregion were categorized according to their inter-observer reliability: those with 0.5 ≤ ICC < 0.75 were classified as moderate, only features with ICC ≥ 0.75 were selected for subsequent model construction.

### 2.7 Model development

To ensure normal distribution and mitigate scale discrepancies among high-dimensional radiomics features, robust features (ICC ≥ 0.75) were normalized using z-score standardization. All subsequent feature selection steps were performed strictly within the training set, without accessing the internal validation set or external test set. Features with a *p*-value > 0.05 in the F-test were then excluded. Subsequently, pairwise correlations were evaluated using Spearman’s rank correlation coefficient; when the correlation between any two features exceeded 0.9, only one was retained. Finally, the Least Absolute Shrinkage and Selection Operator (LASSO) algorithm was applied for further dimensionality reduction. To determine the optimal regularization parameter λ, 5-fold cross-validation was performed strictly within the training set. Following the one-standard-error rule, we selected λ_1se (the largest λ within one standard error of the minimum cross-validated deviance) to favor model sparsity and generalizability. Six machine learning algorithms, including Logistic Regression (LR), Support Vector Machine (SVM), extreme gradient boosting (XGBoost), Decision Tree (DT), Random Forest (RF), and Multilayer Perceptron (MLP), were employed to develop predictive models. The specific default values for each classifier are summarized in Supplementary Table S5. All classifiers were trained on the same training set without additional hyperparameter tuning. The best-performing algorithm was selected for final model development.

### 2.8 K-means habitat analysis for comparison

For comparison, we performed a conventional K-means clustering analysis using the same intratumoral voxel intensities as input. The optimal number of clusters was determined based on the silhouette coefficient. From each K-means habitat, the same set of 107 radiomics features was extracted. Feature reproducibility was assessed with the same image perturbation protocol, retaining only features with ICC ≥ 0.75. The same feature selection and modeling pipeline as used for the Moran’s I habitats was applied to build individual and combined models for the K-means habitats.

### 2.9 Model evaluation on temporal stability

**Fig. 3:**
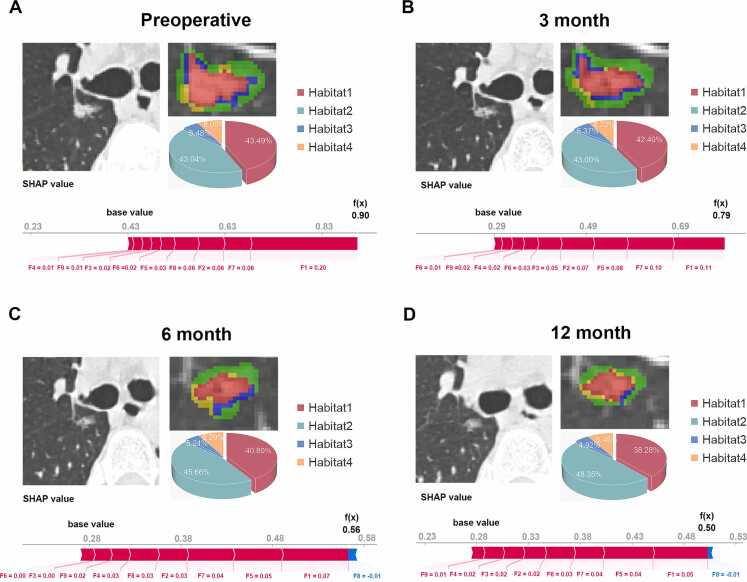
Example prediction for a single patient by the combined model. The pie chart shows the proportional distribution of each habitat, and the bar plot displays the SHAP values for the model's output.

To confirm the temporal stability of the model in pulmonary nodule follow-up management, a comparative analysis was performed using the external testing set with available follow-up CT imaging. All predictions were referenced against the same postoperative pathological diagnosis (the gold standard), as no longitudinal pathological ground truth was available. Thus, this analysis reflects the model's consistency over time rather than prediction of disease progression. Fig. 3 illustrates the longitudinal evolution of habitat distribution for a representative nodule across serial follow-up scans.

### 2.10 Model interpretation

SHapley Additive exPlanations (SHAP) was utilized to interpret the machine learning models, enabling both global and local interpretations of the models. Specifically, global interpretation yielded reliable and consistent attribution values for each feature in the model, thereby reflecting the associations between input features and model outputs.

### 2.11 Statistical analysis

Statistical analyses were performed using Python, version 3.9.0. Continuous variables were compared using ANOVA or the Kruskal-Wallis test; categorical variables using chi-square or Fisher's exact test. Model performance was assessed on both validation set and external testing set. For multiclass evaluation, class-specific AUC values were computed using a one-vs-rest approach. The macro-averaged AUC was derived as the mean of the three class-specific AUCs to provide a class-balance-agnostic summary measure. Additionally, class-wise accuracy, sensitivity, specificity, precision, and F1 score were calculated. DeLong tests were used to compare AUCs between models. A two-sided p-value < 0.05 was considered statistically significant.

## 3 Results

### 3.1 Patients’ characteristics

A total of 614 patients with LUAD were enrolled in this study. Table 1 summarizes the baseline characteristics of patients across the training, validation, and external testing sets. The training set included 400 patients, with pathological diagnoses distributed as follows: 172 cases (43.00%) of AAH/AIS, 115 cases (28.75%) of MIA, and 113 cases (28.25%) of IAC. The validation set included 104 patients, with pathological diagnoses distributed as follows: 44 cases (42.31%) of AAH/AIS, 20 cases (19.23%) of MIA, and 40 cases (38.46%) of IAC. The external testing set included 110 patients, with pathological diagnoses distributed as follows: 37 cases (33.64%) of AAH/AIS, 35 cases (31.82%) of MIA, and 38 cases (34.54%) of IAC. No statistically significant differences were observed among the three cohorts (all *p* values > 0.05), indicating that the groups were well-balanced and comparable.

**Table 1:** Baseline clinical characteristics of the patients in the datasets.

**Variables**	**Training set (n = 400)**	**Validation set (n = 104)**	**External testing set (n = 110)**	***p*****value**
Age	57.31 ± 9.27	56.22 ± 10.01	58.31 ± 9.71	0.273
Sex				0.590
Male	139 (34.75%)	31 (29.81%)	35 (31.81%)	
Female	261 (65.25%)	73 (70.19%)	75 (68.18%)	
Smoking history				0.363
Never	332 (83.00%)	91 (87.50%)	96 (87.27%)	
Former smoker	21 (5.25%)	6 (5.77%)	7 (6.36%)	
Current smoker	47 (11.75%)	7 (6.73%)	7 (6.36%)	
Tumor location				0.950
RUL	154 (38.50%)	39 (37.50%)	39 (35.45%)	
RML	19 (4.75%)	8 (7.69%)	7 (6.36%)	
RLL	71 (17.75%)	15 (14.42%)	17 (15.45%)	
LUL	112 (28.00%)	31 (29.81%)	34 (30.91%)	
LLL	44 (11.00%)	11 (10.58%)	13 (11.82%)	
Pathologic diagnosis				0.154
AAH/AIS	172 (43.00%)	44 (42.31%)	37 (33.64%)	
MIA	115 (28.75%)	20 (19.23%)	35 (31.82%)	
IAC	113 (28.25%)	40 (38.46%)	38 (34.54%)	

Abbreviations: RUL, right upper lobe; RML, right middle lobe; RLL, right lower lobe; LUL, left upper lobe; LLL, left lower lobe; AAH, atypical adenomatous hyperplasia; AIS, adenocarcinoma in situ; MIA, minimally invasive adenocarcinoma; IAC, invasive adenocarcinoma.

For prediction across serial time points, direct comparisons of pathological distributions revealed no significant differences across time points. The pathological diagnoses at each time point were distributed as follows: preoperative, 37 cases (33.64%) of AAH/AIS, 35 cases (31.82%) of MIA, and 38 cases (34.54%) of IAC; at 3 months, 33 cases (33.33%) of AAH/AIS, 31 cases (31.31%) of MIA, and 35 cases (35.35%) of IAC; at 6 months, 36 cases (42.35%) of AAH/AIS, 28 cases (32.94%) of MIA, and 21 cases (24.71%) of IAC; and at 12 months, 37 cases (39.78%) of AAH/AIS, 28 cases (30.11%) of MIA, and 21 cases (30.11%) of IAC (Table S1).

### 3.2 Habitat region and feature selection

**Fig. 4:**
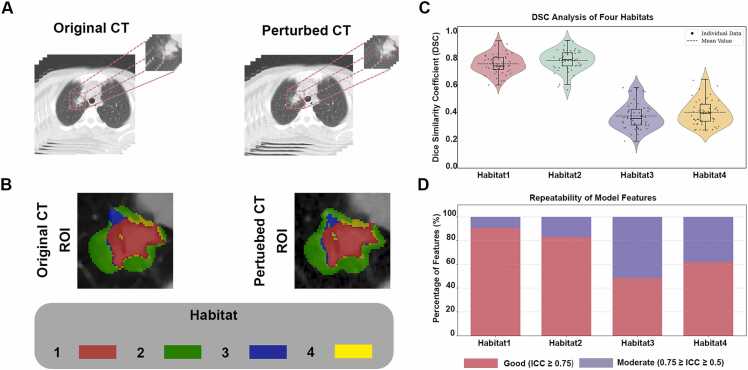
DSC of the habitat regions based on Moran’s I and ICC assessment of radiomics features based on perturbation. (A) Original CT image and CT image after perturbation. (B) Original habitat distribution maps and habitat distribution maps after perturbation. (C) DSC of the four habitat regions. (D) ICC of radiomics features within the four habitat regions.

Following Moran's habitat analysis, the ROIs were divided into four habitat zones. The voxel values and their percentage distributions for the habitats of each pathological finding are presented in **Fig. S1**. Subsequently, two core metrics, DSC and ICC, were evaluated separately: DSC for habitats’ segmentation, and ICC for the extracted features from four habitats. Habitat 1 and Habitat 2 exhibited significantly higher DSC values than Habitat 3 and Habitat 4 (Habiat1: 0.773 ± 0.071 and Habitat2: 0.798 ± 0.078 vs. Habiat3: 0.386 ± 0.096 and Habitat4: 0.415 ± 0.096, *p* < 0.05, Fig. 4**C**). Additionally, a larger proportion of the features in Habitat 1 and Habitat 2 achieved a high ICC grades (90.5%, 82.9% vs. 48.6%, 61.9%, *p* < 0.05, Fig. 4**D**). **Fig. S2** depicts the process of feature selection via the LASSO algorithm across six distinct models, and the features selected through this process were incorporated into subsequent analyses.

### 3.3 Model comparison

**Fig. 5:**
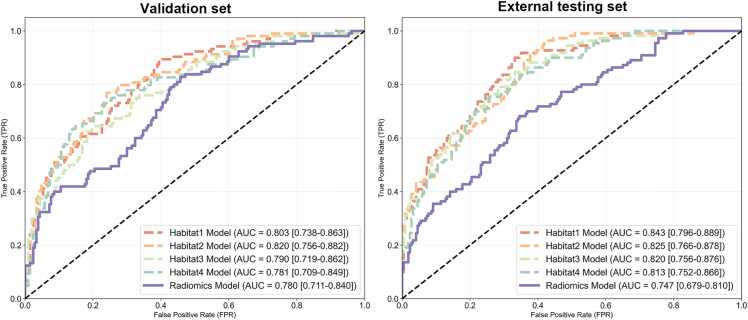
Comparison of the macro-averaged AUC between the habitat model and the radiomics model.

Given the consistent superiority of the SVM model across all evaluated models (Table S3), we primarily report the results derived from the SVM model. The macro-averaged AUC of the Habitat1 model reached 0.803 (95% CI: 0.738, 0.863) in the validation set and 0.843 (95% CI: 0.796, 0.889) in the external testing set, respectively. For the Habitat 2 model, its macro-averaged AUC values were 0.820 (95% CI: 0.756, 0.882) in the validation set and 0.825 (95% CI: 0.766, 0.878) in the external testing set, respectively. Both Habitat1 model and Habitat 2 model demonstrated superior predictive performance compared to the Habitat3 model, Habitat4 model, and Radiomics model (Fig. 5).

**Fig. 6:**
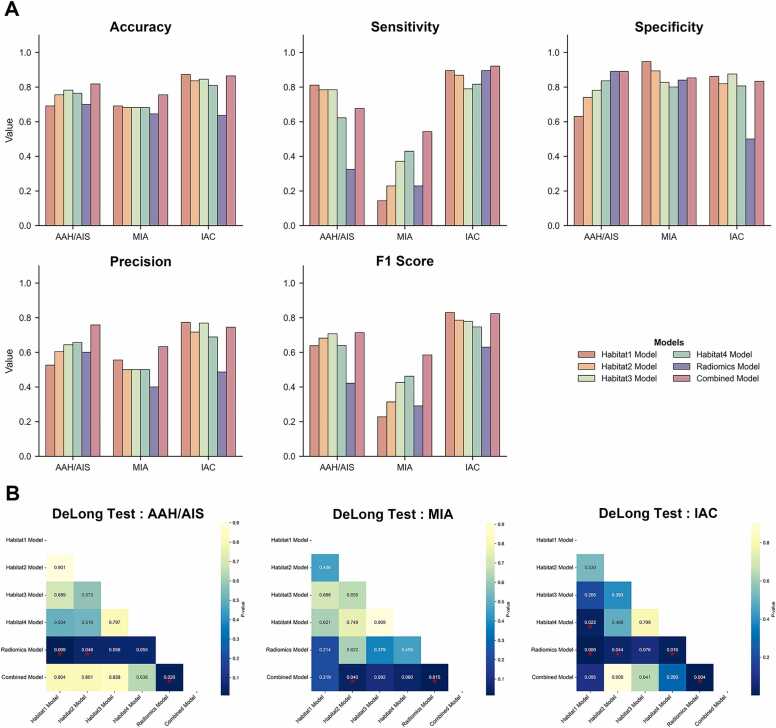
Model performance and statistical comparison. (A) Grouped bar charts of accuracy, sensitivity, specificity, precision, and F1 score for each model across AAH/AIS, MIA, and IAC. (B) DeLong test *p*-values comparing the Combined model with other models for each subtype.

Given the enhanced robustness of features from Habitat 1 and Habitat 2, along with their improved predictive performance, a Combined model integrating these two was developed. Table 2 summarizes the macro-averaged AUC, AUC, accuracy, sensitivity, specificity, precision, and F1 score for all six models on the external testing set. Grouped bar chart illustrating the balance of five diagnostic performance metrics across the models are shown in Fig. 6.

**Table 2:** The performance of predictive models in predicting invasiveness.

**Metrics**	**Validation Set**						**External Testing Set**					
**Performance Measure**	**Combined Model**	**Habitat1 Model**	**Habitat2 Model**	**Habitat3 Model**	**Habitat4 Model**	**Radiomics Model**	**Combined Model**	**Habitat1 Model**	**Habitat2 Model**	**Habitat3 Model**	**Habitat4 Model**	**Radiomics Model**
Macro-averaged AUC	0.830 (0.771, 0.886)	0.803 (0.738, 0.863)	0.820 (0.756, 0.882)	0.790 (0.719, 0.862)	0.781 (0.709, 0.849)	0.780 (0.711, 0.840)	0.854 (0.800, 0.902)	0.843 (0.796, 0.889)	0.825 (0.766, 0.878)	0.820 (0.756, 0.876)	0.813 (0.752, 0.866)	0.747 (0.679, 0.810)
AUC												
AAH/AIS	0.863 (0.784, 0.927)	0.811 (0.692, 0.837)	0.839 (0.751, 0.909)	0.774 (0.655, 0.852)	0.836 (0.725, 0.908)	0.822 (0.757, 0.891)	0.845 (0.751, 0.917)	0.851 (0.773, 0.914)	0.856 (0.794, 0.903)	0.837 (0.758, 0.916)	0.829 (0.768, 0.891)	0.749 (0.654, 0.832)
MIA	0.688 (0.574, 0.790)	0.691 (0.529, 0.697)	0.750 (0.643, 0.852)	0.759 (0.666, 0.838)	0.667 (0.559, 0.749)	0.662 (0.565, 0.789)	0.787 (0.698, 0.865)	0.728 (0.645, 0.826)	0.683 (0.585, 0.757)	0.705 (0.633, 0.812)	0.699 (0.606, 0.779)	0.652 (0.538, 0.759)
IAC	0.939 (0.889, 0.982)	0.906 (0.822, 0.923)	0.871 (0.808, 0.941)	0.837 (0.754, 0.914)	0.840 (0.765, 0.921)	0.857 (0.763, 0.936)	0.931 (0.875, 0.971)	0.951 (0.898, 0.984)	0.935 (0.872, 0.980)	0.919 (0.848, 0.969)	0.911 (0.851, 0.960)	0.840 (0.764, 0.908)
Accuracy												
AAH/AIS	0.798 (0.721, 0.865)	0.654 (0.567, 0.750)	0.731 (0.644, 0.817)	0.673 (0.587, 0.760)	0.760 (0.683, 0.837)	0.505 (0.409, 0.600)	0.818 (0.745, 0.891)	0.691 (0.600, 0.773)	0.755 (0.673, 0.827)	0.782 (0.700, 0.864)	0.764 (0.682, 0.845)	0.700 (0.609, 0.791)
MIA	0.740 (0.663, 0.817)	0.673 (0.587, 0.760)	0.779 (0.692, 0.856)	0.731 (0.635, 0.817)	0.721 (0.635, 0.798)	0.667 (0.571, 0.762)	0.755 (0.673, 0.827)	0.691 (0.609, 0.773)	0.682 (0.591, 0.764)	0.682 (0.600, 0.764)	0.682 (0.591, 0.764)	0.645 (0.555, 0.736)
IAC	0.885 (0.817, 0.942)	0.846 (0.769, 0.913)	0.817 (0.740, 0.885)	0.808 (0.721, 0.885)	0.827 (0.750, 0.894)	0.781 (0.695, 0.857)	0.864 (0.791, 0.927)	0.873 (0.809, 0.936)	0.836 (0.755, 0.900)	0.845 (0.782, 0.909)	0.809 (0.736, 0.882)	0.636 (0.545, 0.727)
Sensitivity												
AAH/AIS	0.727 (0.591, 0.854)	0.846 (0.719, 0.947)	0.846 (0.725, 0.949)	0.795 (0.676, 0.917)	0.821 (0.692, 0.930)	0.974 (0.917, 1.000)	0.676 (0.528, 0.828)	0.811 (0.676, 0.925)	0.784 (0.639, 0.897)	0.784 (0.641, 0.909)	0.622 (0.463, 0.793)	0.324 (0.172, 0.484)
MIA	0.400 (0.182, 0.611)	0.103 (0.000, 0.222)	0.414 (0.241, 0.607)	0.276 (0.121, 0.448)	0.379 (0.208, 0.565)	0.000 (0.000, 0.000)	0.543 (0.367, 0.697)	0.143 (0.029, 0.263)	0.229 (0.094, 0.364)	0.371 (0.214, 0.538)	0.429 (0.267, 0.600)	0.229 (0.095, 0.375)
IAC	0.850 (0.738, 0.954)	0.694 (0.543, 0.833)	0.667 (0.511, 0.813)	0.667 (0.512, 0.811)	0.694 (0.538, 0.838)	0.375 (0.214, 0.542)	0.921 (0.829, 1.000)	0.895 (0.788, 0.974)	0.868 (0.756, 0.970)	0.789 (0.643, 0.900)	0.816 (0.682, 0.935)	0.895 (0.787, 0.975)
Specificity												
AAH/AIS	0.850 (0.758, 0.933)	0.538 (0.422, 0.661)	0.662 (0.550, 0.780)	0.600 (0.484, 0.717)	0.723 (0.614, 0.826)	0.227 (0.131, 0.333)	0.890 (0.819, 0.951)	0.630 (0.521, 0.737)	0.740 (0.628, 0.836)	0.781 (0.675, 0.873)	0.836 (0.747, 0.920)	0.890 (0.812, 0.959)
MIA	0.821 (0.741, 0.904)	0.893 (0.817, 0.957)	0.920 (0.851, 0.974)	0.907 (0.835, 0.972)	0.853 (0.770, 0.928)	0.986 (0.949, 1.000)	0.853 (0.775, 0.929)	0.947 (0.889, 0.987)	0.893 (0.821, 0.957)	0.827 (0.738, 0.908)	0.800 (0.703, 0.892)	0.840 (0.750, 0.916)
IAC	0.906 (0.836, 0.970)	0.926 (0.859, 0.985)	0.897 (0.825, 0.968)	0.882 (0.800, 0.952)	0.897 (0.824, 0.967)	0.959 (0.912, 1.000)	0.833 (0.742, 0.914)	0.861 (0.776, 0.938)	0.819 (0.730, 0.897)	0.875 (0.800, 0.949)	0.806 (0.708, 0.897)	0.500 (0.386, 0.613)
Precision												
AAH/AIS	0.780 (0.643, 0.900)	0.524 (0.397, 0.651)	0.600 (0.472, 0.717)	0.544 (0.417, 0.683)	0.640 (0.510, 0.772)	0.427 (0.322, 0.529)	0.758 (0.611, 0.897)	0.526 (0.392, 0.648)	0.604 (0.458, 0.726)	0.644 (0.500, 0.795)	0.657 (0.500, 0.816)	0.600 (0.385, 0.800)
MIA	0.348 (0.148, 0.556)	0.273 (0.000, 0.539)	0.667 (0.450, 0.889)	0.533 (0.267, 0.800)	0.500 (0.280, 0.714)	0.000 (0.000, 0.000)	0.633 (0.467, 0.808)	0.556 (0.182, 0.857)	0.500 (0.250, 0.765)	0.500 (0.316, 0.700)	0.500 (0.323, 0.680)	0.400 (0.185, 0.611)
IAC	0.850 (0.737, 0.947)	0.833 (0.680, 0.963)	0.774 (0.621, 0.917)	0.750 (0.586, 0.889)	0.781 (0.629, 0.923)	0.800 (0.562, 1.000)	0.745 (0.614, 0.861)	0.773 (0.643, 0.891)	0.717 (0.581, 0.830)	0.769 (0.625, 0.902)	0.689 (0.553, 0.821)	0.486 (0.368, 0.603)
F1 score												
AAH/AIS	0.753 (0.634, 0.845)	0.647 (0.523, 0.745)	0.702 (0.588, 0.800)	0.646 (0.530, 0.755)	0.719 (0.603, 0.813)	0.594 (0.483, 0.691)	0.714 (0.582, 0.828)	0.638 (0.505, 0.740)	0.682 (0.556, 0.769)	0.707 (0.590, 0.818)	0.639 (0.493, 0.760)	0.421 (0.245, 0.567)
MIA	0.372 (0.171, 0.538)	0.150 (0.000, 0.300)	0.511 (0.326, 0.680)	0.364 (0.176, 0.537)	0.431 (0.256, 0.586)	0.000 (0.000, 0.000)	0.585 (0.428, 0.711)	0.227 (0.049, 0.391)	0.314 (0.143, 0.464)	0.426 (0.269, 0.583)	0.462 (0.300, 0.603)	0.291 (0.128, 0.435)
IAC	0.850 (0.757, 0.926)	0.758 (0.618, 0.857)	0.716 (0.588, 0.831)	0.706 (0.567, 0.816)	0.735 (0.610, 0.841)	0.511 (0.326, 0.667)	0.824 (0.725, 0.906)	0.829 (0.732, 0.909)	0.786 (0.682, 0.868)	0.779 (0.657, 0.874)	0.747 (0.634, 0.841)	0.630 (0.511, 0.733)

Numbers in parentheses are 95% confidence intervals via bootstrap. Abbreviations: AAH, atypical adenomatous hyperplasia; AIS, adenocarcinoma in situ; MIA, minimally invasive adenocarcinoma; IAC, invasive adenocarcinoma; AUC, area under the receiver operating characteristic curve.

DeLong tests confirmed that the Combined model significantly outperformed the conventional Radiomics model across all three classes: AAH/AIS (0.845 vs. 0.749, *p* = 0.020), MIA (0.787 vs. 0.652, *p* = 0.015), and IAC (0.931 vs. 0.840, *p* = 0.004).

### 3.4 Model performance

**Fig. 7:**
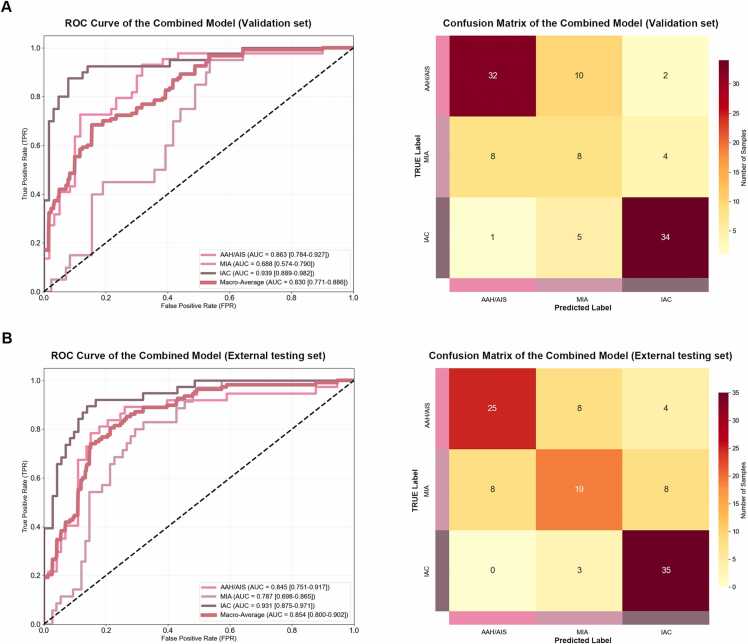
Performance of the combined model in the three-class classification task. (A) Validation set results. (B) External testing set results.

The Combined model was identified as the optimal model. Fig. 7**A–B** illustrates its performance on the validation set and external testing set, with its macro-averaged AUC values reaching 0.830 (95% CI: 0.771, 0.886) and 0.854 (95% CI: 0.800, 0.902), corresponding to the two sets respectively. Fig. 7**C–D** presents the heatmaps of the confusion matrices for the Combined model’s ternary classification, which visualize the discriminative results between predicted labels and true labels on the validation set and external testing set.

### 3.5 Comparison with K-means habitat analysis

We compared the Moran’s I-driven habitat approach with the K-means algorithm and the results are summarized in Fig. S3. In terms of habitat robustness, Moran’s I habitats (Habitat1 and Habitat2) were significantly more stable under image perturbation than the three K-means habitats (DSC: 0.382, 0.355, 0.323; all *p* < 0.001). Regarding feature reproducibility, Moran’s I habitats retained 90.5% (Habitat1) and 82.9% (Habitat2) of radiomics features with ICC ≥ 0.75, whereas K-means habitats retained only 30.8%, 26.2%, and 37.4%. For model performance, the Moran’s I combined model achieved a macro-averaged AUC of 0.854 in the external test set, higher than that of the K-means combined habitat model (0.730). DeLong tests showed that the Moran’s I model significantly outperformed the K-means model for MIA (0.787 vs. 0.545, *p* < 0.001) and IAC (0.931 vs. 0.904, *p* = 0.001), while both performed comparably for AAH/AIS (0.845 vs. 0.814, *p* = 0.344).

### 3.6 Model interpretation

**Fig. 8:**
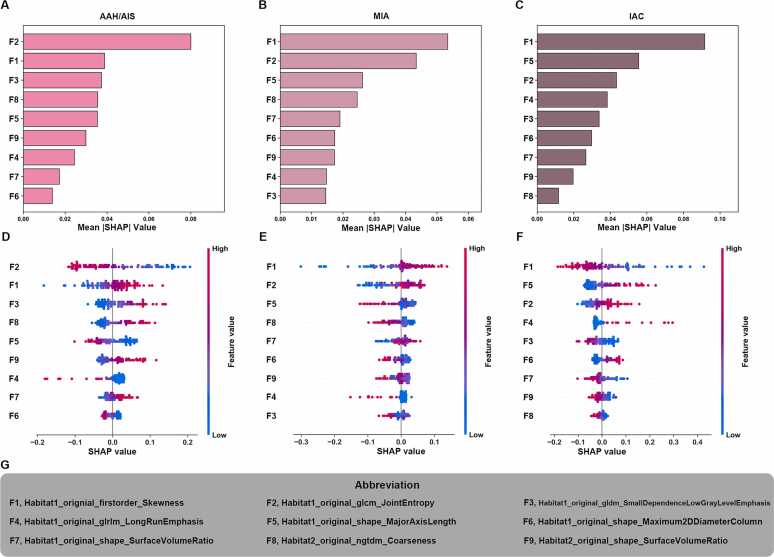
Feature importance and SHAP analysis. (A-C) Feature importance rankings for predicting AAH/AIS, MIA, and IAC. (D-F) SHAP beeswarm plots for AAH/AIS, MIA, and IAC. (G) List and descriptions of the radiomics features.

The global explanation captures the overall behavior of the model. According to the SHAP analysis results (Fig. 8), features F1 (*Habitat 1_orignial_firstorder_Skewness*), F2 (*Habitat 1_original_glcm_JointEntropy*), and F5 (*Habitat 1_original_shape_MajorAxisLength*) emerged as the most influential contributors. Specifically, F2 serves as the most important feature in predicting AAH and has a negative impact on the model’s output for this class; conversely, it exerts a positive influence in predicting MIA and IAC. For MIA and IAC predictions, F1 is the most critical feature: it positively contributes to MIA prediction but negatively affects IAC prediction. In contrast, F5 shows an opposing pattern: it has a positive effect on IAC prediction, while negatively influencing predictions for both AAH and MIA. Fig. S4 illustrates the correspondence between habitat subregions and histopathological findings at the plane of maximum tumor diameter in three representative CT cases.

### 3.7 Prediction consistency across serial time points

**Fig. 9:**
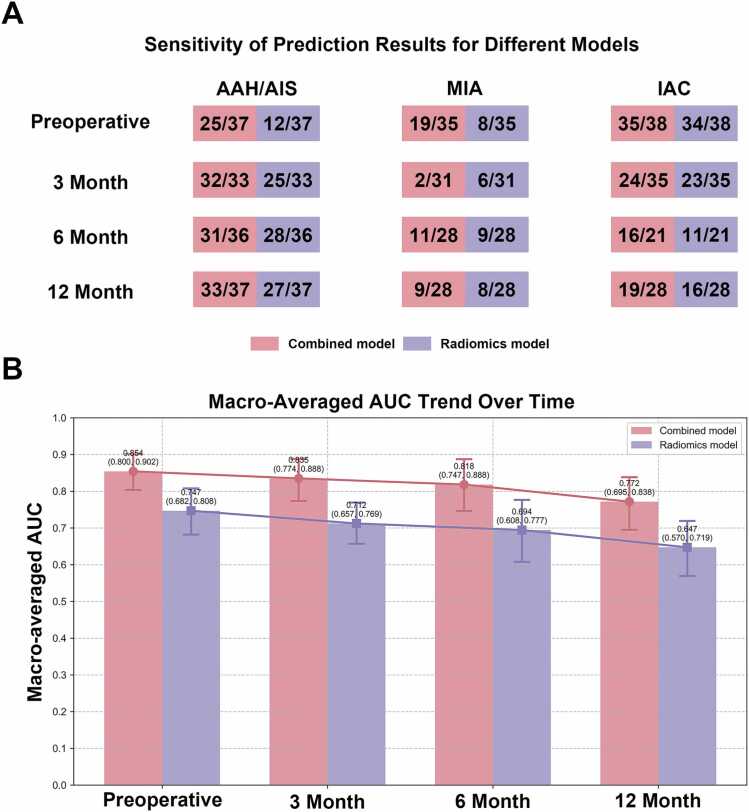
Temporal prediction performance comparison between the combined model and the radiomics model. (A) Sensitivity. (B) Macro-averaged AUC.

In the serial preoperative CT analysis, the Combined model exhibited superior sensitivity compared to the conventional Radiomics model. This advantage was particularly pronounced for identifying IAC, where the Combined model correctly identified more cases across all preoperative time points: preoperative (35/38 vs. 34/38), 3 months (24/35 vs. 23/35), 6 months (16/21 vs. 11/21), and 12 months (19/28 vs. 16/28) (Fig. 9**A**). Furthermore, the Combined model consistently achieved higher macro-averaged AUCs throughout the longitudinal observation period (Fig. 9**B**).

## 4 Discussion

This study established a biologically plausible and technically robust habitat radiomics model for the preoperative stratification of LUAD invasiveness. Addressing the inherent limitations of conventional radiomics in masking intratumoral heterogeneity and the stochastic instability of unsupervised clustering, our Moran’s I-driven approach spatially dissected nodules into distinct phenotypic habitats. The Combined model not only achieved a superior macro-averaged AUC of 0.854 (95% CI: 0.800–0.902) for three-class classification in the external testing set, but also demonstrated good temporal consistency across longitudinal scans. These findings, combined with consistent performance across serial preoperative scans, establish the model as a temporally stable and biological plausible tool for preoperative clinical decision-making.

The Combined model achieved a class-specific AUC of 0.845 (95% CI: 0.751, 0.917) and 0.787 (95% CI: 0.698, 0.865) in predicting AAH/AIS and MIA, respectively. More importantly, as IAC is a category with poor prognosis, the Combined model yielded an AUC of 0.931 (95% CI: 0.875, 0.971) when predicting IAC, which exceeded the previously reported AUC range (0.732–0.836) of conventional radiomics models [20], [21], [22]. These results indicate that our habitat model, which captures distinct morphological regions (e.g., high-density core [Habitat1] and invasive ground-glass margin [Habitat2]), outperforms conventional approaches. Moreover, the Combined model attained a macro-averaged AUC of 0.854, surpassing the macro-averaged AUC of 0.772–0.802 reported in two other ITH-based habitat studies [23], [24]. In our comparison with K-means clustering under the same pipeline, the Moran’s I-based combined model achieved a markedly higher macro-averaged AUC, with significant improvements for MIA and IAC. These findings collectively underscore the superior predictive capability of Moran’s I-based radiomics.

Robustness of the diagnostic model is a prerequisite for clinical translation. Current habitat studies predominately use unsupervised clustering such as K-means, which suffers from initialization randomness and moderate reproducibility (DSC: 0.424–0.712) [17]. In our direct comparison under the same perturbation protocol, conventional K-means achieved even lower DSC values (0.323–0.382). In contrast, our Moran’s I habitats yielded significantly higher clustering stability (Habitat1: 0.773; Habitat2: 0.798). Moreover, the repeatability of radiomics features was markedly better for Moran’s I habitats: 90.5% and 82.9% of features from Habitat1 and Habitat2, respectively, had ICC ≥ 0.75, whereas K-means habitats retained only 26–37% of robust features. Of the nine features in the final model, eight were confirmed to be highly repeatable [18]. The feature *Habitat1_orignial_firstorder_Skewness* likely maintained high ICC in our subregions, as our method is based on spatial correlation.

A distinctive strength of this study is the validation of temporal stability. The clinical management of indeterminate pulmonary nodules often relies on active surveillance using low dose CT screening [25], [26]. Our study evaluated a screening cohort with longitudinal CT screening and demonstrated that the combined model achieved macro-averaged AUC from 0.772 to 0.854 across all time points. This performance consistently surpassed the conventional radiomics model (macro-averaged AUC: 0.647–0.747), indicating superior temporal stability in predictive performance. Notably, the Combined Model identified more IAC cases than conventional radiomics model during pulmonary nodule follow-up screening. This indicates its ability to decode heterogeneous changes in early-stage LUAD [27]. Furthermore, observed prediction downgrades in some nodules may be attributed to inconsistent inspiratory amplitude during scanning, highlighting a critical technical factor in longitudinal screening management [28].

The superior predictive performance of our model may be partly attributed to its ability to capture spatial heterogeneity that is biologically plausible. SHAP analysis revealed that texture features within Habitat 1 were the strongest predictors. Specifically, *Habitat1_original_firstorder_Skewness*, which reflects the skewness of the gray-level distribution in Habitat 1, exhibited a positive influence on the prediction of AAH/AIS and MIA, but shifted to a negative effect in predicting IAC. This pattern is consistent with the hypothesis that pre-invasive lesions have more heterogeneous density distributions, while invasive cancers may exhibit more homogeneous dense cellular aggregation [29], [30]. However, direct histopathological validation is needed to confirm this interpretation. Similarly, *Habitat1_original_glcm_JointEntropy* captures the higher-order gray-level distribution within Habitat1. It demonstrated a positive effect in predicting AAH/AIS and was also positively associated with distinguishing MIA from IAC. This observation aligns with the understanding that cellular density in the tumor’s central region serves as a crucial determinant of invasiveness in pulmonary nodules [31]. Regarding morphological features, compared to AAH/AIS and MIA, IAC often presents with an increased radius in both Habitat 1 and Habitat 2. Consequently, an increased nodule radius is a key morphological indicator for predicting IAC [32], [33].

Our study has limitations. First, the retrospective design necessitates future large-scale prospective validation to confirm generalizability. Second, while our habitat interpretations (e.g., core vs. invasive front) are biologically plausible, they remain speculative without direct histopathological co-registration or spatial transcriptomic validation. Future studies with paired imaging and pathological mapping are needed to verify these biological assignments. Third, the discrimination performance for MIA was modest (AUC 0.787, 54% accuracy). This likely reflects the small MIA sample size in the external testing set and the inherent histologic heterogeneity of MIA, and it underscores the need for larger MIA-focused studies. Fourth, the external cohort required at least three serial preoperative CT scans, which introduced a selection bias toward slow-growing, indolent nodules. Therefore, the model’s performance on rapidly progressive or more aggressive tumors remains unknown, limiting generalizability to such lesions. Finally, manual ROI segmentation is inherently subjective, and we did not quantitatively assess inter-observer reproducibility. Replacing manual segmentation with deep learning-based automation in future studies would enhance objectivity, efficiency, and reproducibility.

## 5 Conclusions

In this study, we developed a radiomics model based on Moran’s habitat analysis. For the differentiation of postoperative AAH/AIS, MIA, and IAC of LUAD, this model achieved a macro-averaged AUC of 0.854 (95% CI: 0.800–0.902) in the external dataset. Compared with conventional radiomics, the proposed model exhibited more accurate non-invasive prediction of postoperative pathological subtypes of pulmonary nodules and more reliable follow-up management of these nodules. The integration of biological plausible and temporal stable performance in the habitat model supports its clinical utility for non-invasive risk stratification of pulmonary nodules.

## CRediT authorship contribution statement

**Fajin Lv:** Writing – review & editing, Funding acquisition. **Xiaogang Chen:** Data curation. **Sifan Chen:** Writing – original draft, Data curation, Conceptualization. **Maolu Tan:** Supervision. **Bo Li:** Data curation. **Lingqi Gao:** Writing – original draft, Visualization, Methodology, Conceptualization.

## Ethical statement

The study protocol was approved by the institutional ethics committee (IRB, No. 2024-046-01), and the requirement for informed consent was waived due to the use of anonymized data and the retrospective nature of the study.

## Funding

This work was supported by the Chongqing Health Appropriate Technology Promotion Project (No. 2023jstg044) and the Joint Project of Chongqing Health Commission and Science and Technology Bureau (No. 2022ZDXM006).

## Declaration of Competing Interest

The authors declare that they have no known competing financial interests or personal relationships that could have appeared to influence the work reported in this paper.
