# Supplementary material for: Moran's I-driven habitat radiomics: A biologically plausible and temporally robust approach for risk stratification of lung adenocarcinoma invasiveness
Source: Eur J Radiol Open. 2026 Jul 14;17:100792. doi: 10.1016/j.ejro.2026.100792 (PMC13377481; doi:10.1016/j.ejro.2026.100792)
Supplement: Supplementary file 1 — Supplementary material [file mmc1.docx]

Fig. S1 Path diagrams, mean squared errors, and coefficients of LASSO regression in the Combined model, Habitat1 model, Habitat2 model, Habitat3 model, Habitat4 model, and Radiomics model (A-F) Results corresponding to Combined model, Habitat1 model, Habitat2 model, Habitat3 model, Habitat4 model, and Radiomics model respectively.

Fig. S2 Voxel values and proportion of the four types of habitats (Habitat1, Habitat2, Habitat3, and Habitat4) among three categories of nodules (AAH/AIS, MIA, and IAC). (A) Voxel values of the four habitats across the three nodule categories. (B) Proportion of the four habitats across the three nodule categories.

Fig. S3. Comparison between Moran’s I‑driven habitats and K‑means clustering (k=3). (A) DSC of K‑means habitats. (B) Percentage of robust features per K‑means habitat. (C) Macro‑AUC of K‑means habitat models. (D) Class‑specific AUCs for the K‑means combined model. (E) DSC comparison. (F) DeLong test: Moran’s I combined model vs. K‑means combined model for AAH/AIS MIA, and IAC.

Fig. S4 Comparison of CT images, habitat subregion maps, and histopathological whole‑mount sections (maximum tumor diameter level) for three representative nodules.
