# Supplementary material for: Moran's I-driven habitat radiomics: A biologically plausible and temporally robust approach for risk stratification of lung adenocarcinoma invasiveness
Source: Eur J Radiol Open. 2026 Jul 14;17:100792. doi: 10.1016/j.ejro.2026.100792 (PMC13377481; doi:10.1016/j.ejro.2026.100792)
Supplement: Supplementary file 2 — Supplementary material [file mmc2.docx]

**Supplementary Materials**

**Supplementary Appendix 1**

Radiomics Quality Score (RQS)

RQS was put forward by the radiomics community as a comprehensive score that reflects the methodological quality of a radiomics study. This score ranges from -8 to 36, and is calculated by summing up the ratings across 16 dimensions—each corresponding to a key component in the radiomics workflow, including imaging protocol quality and model validation.We evaluated the quality of our own study and achieved an RQS score of 17 (equivalent to 47.22%).

1. Image protocol quality: protocols well documented (Table S2)
2. Multiple segmentations and perturbing segmentations: yes (Manuscript: Methods, Image perturbed and feature extraction)
3. Phantom study or analysis of feature robustness: yes (Manuscript: Methods, Repeatability Analyses to Identify Precise Feature)
4. Imaging at multiple timepoints: yes (Manuscript: Methods, Time Serial Prediction)
5. Feature reduction methods: either measure is implemented (Manuscript: Methods, Radiomics Feature Selection and Model Development and Supplementary Figure S1)
6. Multivariable analysis with non-radiomic features: no
7. Biological correlates: no
8. Cut-off analysis: no
9. Discrimination statistics: discrimination statistics and its significance are reported, resampling technique is also applied (Manuscript: Methods, Radiomics Feature Selection and Model Development)
10. Calibration analysis: calibration statistic and its significance are reported, a resampling method is applied (Manuscript: Methods, Radiomics Feature Selection and Model Development and Statistical Analysis)
11. Prospective study: no
12. Validation: based on a dataset from another institute (Manuscript: Methods, Patients’ Characteristics)
13. Comparison to ‘gold standard’: yes (Manuscript: Methods, The patients have postoperative pathology.)
14. Potential clinical utility: no
15. Cost-effectiveness analysis: no
16. Open science and data: scans are open source, code is open source (Manuscript: Methods, Patients’ Characteristics. codes repository: https://github.com/410312774/PixelMedAI)

**Supplementary Appendix 2**

Details of Moran’s Habitat Analysis

### 1. Basic Math Definitions

For an ROI in CT images (3D voxel set $\text{\{}\text{x}_{\text{i}}\text{∣i=1,…,n\}}$, $\text{x}_{\text{i}}$=voxel attribute value, $\text{n}$=total voxels):

- Regional mean: $\text{x}\text{=}\frac{\text{1}}{\text{n}}\sum_{\text{i=1}}^{\text{n}} \text{x}_{\text{i}}$
- Regional variance (standardization factor): $M_{2}=\frac{1}{n}\sum_{i=1}^{n} (x_{i}-\overline{x})^{2}$

### 2. Spatial Weight Matrix ($\text{W=}\left[ \text{w}_{\text{ij}} \right]_{\text{n×n}}$)

Quantifies voxel neighborhood via 3D Euclidean distance:

- Distance between voxel $\text{i}\left( \text{x}_{\text{i}}\text{,}\text{y}_{\text{i}}\text{,}\text{z}_{\text{i}} \right)$ and $\text{j}\left( \text{x}_{\text{j}}\text{,}\text{y}_{\text{j}}\text{,}\text{z}_{\text{j}} \right)$: $\text{d}_{\text{ij}}\text{=}\sqrt{\left( \text{x}_{\text{i}}\text{−}\text{x}_{\text{j}} \right)^{\text{2}}\text{+}\left( \text{y}_{\text{i}}\text{−}\text{y}_{\text{j}} \right)^{\text{2}}\text{+}\left( \text{z}_{\text{i}}\text{−}\text{z}_{\text{j}} \right)^{\text{2}}}$
- Rule: $\text{w}_{\text{ij}}\text{=1}$ if $\text{d}_{\text{ij}}\text{≤}\sqrt{\text{3}}$ (8-connected 3D neighborhood), else $\text{w}_{\text{ij}}\text{=0}$
- Property: Symmetric sparse matrix ($\text{w}_{\text{ij}}\text{=}\text{w}_{\text{ji}}$, $\text{w}_{\text{ii}}\text{=0}$)

### 3. Local Moran’s Index ($\text{I}_{\text{i}}$)

Measures voxel spatial correlation:

$$I_{i}=\frac{(x_{i}-\overline{x})}{M_{2}}\sum_{j} w_{ij}(x_{j}-\overline{x})$$

Where: $\text{x}_{\text{j}}$=voxel $\text{j}$ attribute, $\text{x}$=regional mean, $\text{M}_{\text{2}}$=regional variance, $\text{w}_{\text{ij}}$=weight matrix element

### 4. Habitat Classification Criteria

Based on 3 factors: $\left| \text{I}_{\text{i}} \right|$ (significance), $\text{I}_{\text{i}}$ direction, $\text{x}_{\text{i}}$ vs. $\text{x}$ ($\text{I}_{\text{α}}$=critical value at $\text{α=0.05}$):

| Habitat Category | Judgment Condition |
| --- | --- |
| 1 Habitat1 (High-High Clustering) | $\text{I}_{\text{i}}\text{>0}$, $\left\vert\text{I}_{\text{i}} \right\vert\text{>}\text{I}_{\text{α}}$, $\text{x}_{\text{i}}\text{>}\text{x}$ |
| 2 Habitat2 (Low-Low Clustering) | $\text{I}_{\text{i}}\text{>0}$, $\left\vert\text{I}_{\text{i}} \right\vert\text{>}\text{I}_{\text{α}}$, $\text{x}_{\text{i}}\text{<}\text{x}$ |
| 3 Habitat3 (High-Low Outlier) | $\text{I}_{\text{i}}\text{<0}$, $\left\vert\text{I}_{\text{i}} \right\vert\text{>}\text{I}_{\text{α}}$, $\text{x}_{\text{i}}\text{>}\text{x}$ |
| 4 Habitat4 (Low-High Outlier) | $\text{I}_{\text{i}}\text{<0}$, $\left\vert\text{I}_{\text{i}} \right\vert\text{>}\text{I}_{\text{α}}$, $\text{x}_{\text{i}}\text{<}\text{x}$ |

### 5. PixelMed AI Platform Parameters

- Significance level: $\text{α=0.05}$ ($\text{I}_{\text{0.05}}\text{≈1.96}$ for large $\text{n}$)
- Distance threshold: $\sqrt{\text{3}}$ (8-connected neighborhood)
- Voxel normalization: $\text{x}_{\text{i}}\text{′=}\frac{\text{x}_{\text{i}}\text{−min}\left( \text{x} \right)}{\text{max}\left( \text{x} \right)\text{−min}\left( \text{x} \right)}$ ($\text{min}\left( \text{x} \right)$, $\text{max}\left( \text{x} \right)$=ROI attribute bounds

**Supplementary Tables**

**Table S1** Baseline clinical characteristics of the patients in time serial prediction.

| **Variables** | **Preoperative (n = 110)** | **3 month (n = 99)** | **6 month (n = 85)** | **12month (n = 93)** | ***p* value** |
| --- | --- | --- | --- | --- | --- |
| Age | 58.31 ± 9.71 | 57.21 ± 9.64 | 57.69 ± 9.87 | 56.91 ± 9.91 |  |
| Sex |  |  |  |  | 0.928 |
| Male | 35 (31.81%) | 29 (29.29%) | 24 (28.24%) | 26 (27.96%) |  |
| Female | 75 (68.18%) | 70 (70.71%) | 61 (71.76%) | 67 (72.04%) |  |
| Smoking history |  |  |  |  | 0.998 |
| Never | 96 (87.27%) | 87 (87.88%) | 75 (88.24%) | 81 (87.10%) |  |
| Former smoker | 7 (6.36%) | 6 (6.06%) | 4 (4.71%) | 5 (5.38%) |  |
| Current smoker | 7 (6.36%) | 6 (6.06%) | 6 (7.06%) | 7 (7.53%) |  |
| Tumor location |  |  |  |  | 1.000 |
| RUL | 39 (35.45%) | 37 (37.37%) | 32 (37.65%) | 36 (38.71%) |  |
| RML | 7 (6.36%) | 6 (6.06%) | 4 (4.71%) | 5 (5.38%) |  |
| RLL | 17 (15.45%) | 14 (14.14%) | 13 (15.29%) | 14 (15.05%) |  |
| LUL | 34 (30.91%) | 32 (32.32%) | 27 (31.76%) | 30 (32.26%) |  |
| LLL | 13 (11.82%) | 10 (10.10%) | 9 (10.59%) | 8 (8.60%) |  |
| Pathologic diagnosis |  |  |  |  | 0.709 |
| AAH/AIS | 37 (33.64%) | 33 (33.33%) | 36 (42.35%) | 37 (39.78%) |  |
| MIA | 35 (31.82%) | 31 (31.31%) | 28 (32.94%) | 28 (30.11%) |  |
| IAC | 38 (34.54%) | 35 (35.35%) | 21 (24.71%) | 28 (30.11%) |  |

Abbreviations: RUL, right upper lobe; RML, right middle lobe; RLL, right lower lobe; LUL, left upper lobe; LLL, left lower lobe; AAH, atypical adenomatous hyperplasia; AIS, adenocarcinoma in situ; MIA, minimally invasive adenocarcinoma; IAC, invasive adenocarcinoma.

**Table S2** CT scan and reconstruction parameters.

|  | Center A | | | Center B | |
| --- | --- | --- | --- | --- | --- |
| Machine | GE Discovery 750HD | GE Optima 660 | GE Revolution | Siemens AG SOMATOM | GE Discovery 750HD |
| Tube voltage (kV) | 120 | 120 | 120 | 120 | 120 |
| Tube current (mA) | Variable tube current with ATCM | Variable tube current with ATCM | Variable tube current with ATCM | Variable tube current with ATCM | Variable tube current with ATCM |
| Rotation time (s) | 0.6 | 0.5 | 0.5 | 0.5 | 0.5 |
| Pitch | 0.984 | 0.984 | 0.992 | 1.2 | 0.984 |
| Thickness (mm) | 1.25 | 1.25 | 1.25 | 1 | 0.625 |
| Interval (mm) | 1.25 | 1.25 | 1.25 | 1 | 0.625 |
| Kernel | Lung | Lung | Lung | Br40 | Lung |
| Reconstruction algorithm | Asir-30 | Asir-30 | Asir-10 | IR3 | Asir-30 |
| Matrix | 512 × 512 | 512 × 512 | 512 × 512 | 512 × 512 | 512 × 512 |

Abbreviations: kV, kilovolt; mA, milliampere; mm, millimeter. ATCM, automatic tube current modulation

**Table S3** Source of extracted features derived from pyradiomics.

| **Feature classes** | **Feature names** | **Number of features** |
| --- | --- | --- |
| Shape-based | Elongation,Flatness,LeastAxisLength,MajorAxisLength,Maximum2DDiameterColumn,Maximum2DDiameterRow,Maximum2DDiameterSlice,Maximum3DDiameter,MeshVolume,MinorAxisLength,Sphericity,SurfaceArea,SurfaceVolumeRatio,VoxelVolume | (14 features) |
| First Order Statistics | 10Percentile,90Percentile, Energy, Entropy, InterquartileRange, Kurtosis, Maximum, MeanAbsoluteDeviation, Mean, Median, Minimum, Range, RobustMeanAbsoluteDeviation, RootMeanSquared, Skewness, TotalEnergy, Uniformity, Variance | (18 features) |
| Grey Level Cooccurrence Matrix | Autocorrelation,ClusterProminence,ClusterShade,ClusterTendency,Contrast,Correlation,DifferenceAverage,DifferenceEntropy,DifferenceVariance,Id,Idm,Idmn,Idn,Imc1,Imc2,InverseVariance,JointAverage,JointEnergy,JointEntropy,MCC,MaximumProbability,SumAverage,SumEntropy,SumSquares | (glcm,24 features) |
| Grey Level Size Zone Matrix | GrayLevelNonUniformity,GrayLevelNonUniformityNormalized,GrayLevelVariance,HighGrayLevelZoneEmphasis,LargeAreaEmphasis,LargeAreaHighGrayLevelEmphasis,LargeAreaLowGrayLevelEmphasis,LowGrayLevelZoneEmphasis,SizeZoneNonUniformity,SizeZoneNonUniformityNormalized,SmallAreaEmphasis,SmallAreaHighGrayLevelEmphasis,SmallAreaLowGrayLevelEmphasis,ZoneEntropy,ZonePercentage,ZoneVariance,Busyness,Coarseness,Complexity,Contrast,Strength | (glszm,21 features) |
| Grey Level Run Length Matrix | GrayLevelNonUniformity,GrayLevelNonUniformityNormalized,GrayLevelVariance,HighGrayLevelRunEmphasis,LongRunEmphasis,LongRunHighGrayLevelEmphasis,LongRunLowGrayLevelEmphasis,LowGrayLevelRunEmphasis,RunEntropy,RunLengthNonUniformity,RunLengthNonUniformityNormalized,RunPercentage,RunVariance,ShortRunEmphasis,ShortRunHighGrayLevelEmphasis,ShortRunLowGrayLevelEmphasis | (glrlm,16 features) |
| Grey Level Dependence Matrix | DependenceEntropy,DependenceNonUniformity,DependenceNonUniformityNormalized,DependenceVariance,GrayLevelNonUniformity,GrayLevelVariance,HighGrayLevelEmphasis,LargeDependenceEmphasis,LargeDependenceHighGrayLevelEmphasis,LargeDependenceLowGrayLevelEmphasis,LowGrayLevelEmphasis,SmallDependenceEmphasis,SmallDependenceHighGrayLevelEmphasis,SmallDependenceLowGrayLevelEmphasis | (gldm,14 features) |

**Table S4** Comparison of model performance among different machine learning algorithms.

| Macro-average AUC | Combined Model | Habitat1 Model | Habitat2 Model | Habitat3 Model | Habitat4 Model | Radiomics Model |
| --- | --- | --- | --- | --- | --- | --- |
| Training set |  |  |  |  |  |  |
| LR | 0.865 (0.839, 0.894) | 0.865 (0.838, 0.890) | 0.844 (0.816, 0.873) | 0.822 (0.792, 0.852) | 0.859 (0.829, 0.885) | 0.863 (0.835, 0.890) |
| SVM | 0.894 (0.871, 0.917) | 0.902 (0.879, 0.923) | 0.890 (0.866, 0.912) | 0.856 (0.827, 0.884) | 0.896 (0.871, 0.918) | 0.888 (0.863, 0.913) |
| DT | 1.000 (1.000, 1.000) | 1.000 (1.000, 1.000) | 1.000 (1.000, 1.000) | 1.000 (1.000, 1.000) | 1.000 (1.000, 1.000) | 1.000 (1.000, 1.000) |
| RF | 1.000 (1.000, 1.000) | 1.000 (1.000, 1.000) | 1.000 (1.000, 1.000) | 1.000 (1.000, 1.000) | 1.000 (1.000, 1.000) | 1.000 (1.000, 1.000) |
| XGBOOST | 1.000 (1.000, 1.000) | 1.000 (1.000, 1.000) | 1.000 (1.000, 1.000) | 1.000 (1.000, 1.000) | 1.000 (1.000, 1.000) | 1.000 (1.000, 1.000) |
| MLP | 0.983 (0.976, 0.990) | 0.988 (0.980, 0.993) | 0.769 (0.703, 0.828) | 0.917 (0.897, 0.937) | 0.999 (0.999, 0.999) | 1.000 (1.000, 1.000) |
| Validation set |  |  |  |  |  |  |
| LR | 0.824 (0.757, 0.884) | 0.801 (0.752, 0.882) | 0.814 (0.754, 0.878) | 0.792 (0.721, 0.862) | 0.771 (0.703, 0.830) | 0.729 (0.658, 0.787) |
| SVM | 0.830 (0.771, 0.886) | 0.803 (0.756, 0.882) | 0.820 (0.756, 0.882) | 0.790 (0.719, 0.862) | 0.781 (0.709, 0.849) | 0.780 (0.711, 0.840) |
| DT | 0.643 (0.575, 0.710) | 0.759 (0.693, 0.828) | 0.640 (0.565, 0.709) | 0.682 (0.616, 0.753) | 0.663 (0.599, 0.728) | 0.599 (0.530, 0.673) |
| RF | 0.813 (0.744, 0.875) | 0.789 (0.714, 0.864) | 0.805 (0.737, 0.867) | 0.792 (0.716, 0.853) | 0.817 (0.749, 0.883) | 0.766 (0.700, 0.831) |
| XGBOOST | 0.798 (0.731, 0.865) | 0.791 (0.727, 0.851) | 0.784 (0.708, 0.847) | 0.774 (0.697, 0.842) | 0.770 (0.691, 0.839) | 0.754 (0.681, 0.828) |
| MLP | 0.767 (0.698, 0.837) | 0.791 (0.717, 0.859) | 0.814 (0.747, 0.878) | 0.803 (0.733, 0.865) | 0.779 (0.706, 0.849) | 0.744 (0.670, 0.812) |
| External testing set |  |  |  |  |  |  |
| LR | 0.833 (0.777, 0.884) | 0.831 (0.788, 0.890) | 0.793 (0.731, 0.851) | 0.815 (0.752, 0.870) | 0.791 (0.724, 0.850) | 0.685 (0.609, 0.760) |
| SVM | 0.854 (0.800, 0.902) | 0.843 (0.796, 0.889) | 0.825 (0.766, 0.878) | 0.820 (0.756, 0.876) | 0.813 (0.752, 0.866) | 0.747 (0.679, 0.810) |
| DT | 0.682 (0.621, 0.747) | 0.725 (0.655, 0.794) | 0.691 (0.625, 0.755) | 0.670 (0.601, 0.735) | 0.671 (0.604, 0.743) | 0.629 (0.567, 0.689) |
| RF | 0.850 (0.798, 0.895) | 0.826 (0.780, 0.885) | 0.794 (0.733, 0.856) | 0.792 (0.726, 0.852) | 0.802 (0.736, 0.858) | 0.666 (0.595, 0.739) |
| XGBOOST | 0.831 (0.774, 0.883) | 0.835 (0.775, 0.890) | 0.777 (0.714, 0.837) | 0.781 (0.714, 0.845) | 0.802 (0.738, 0.863) | 0.721 (0.656, 0.786) |
| MLP | 0.817 (0.757, 0.874) | 0.771 (0.704, 0.833) | 0.769 (0.706, 0.833) | 0.812 (0.754, 0.868) | 0.807 (0.741, 0.869) | 0.732 (0.660, 0.792) |

Abbreviations: LR, logistic regression; SVM, support vector machine; DT, decision tree, RF, random forest, XGBoost, extreme gradient boosting, MLP, multilayer perceptron. AUC, area under the receiver operating characteristic curve.

**Table S5** the final parameter settings for each classifier

| **Classifier** | **Package / Version** | **Key hyperparameters** | **Other parameters** |
| --- | --- | --- | --- |
| LR | scikit-learn 1.2.0 | random_state=0 | C=1.0, penalty='l2', solver='lbfgs', max_iter=100 |
| SVM | scikit-learn 1.2.0 | kernel='rbf', class_weight='balanced', probability=True, max_iter=1000 | C=1.0, gamma='scale' |
| XGBoost | xgboost 1.7.0 | class_weight='balanced' | n_estimators=100, max_depth=6, learning_rate=0.3, subsample=1.0 |
| DT | scikit-learn 1.2.0 | class_weight='balanced' | criterion='gini', max_depth=None, min_samples_split=2, min_samples_leaf=1 |
| RF | scikit-learn 1.2.0 | class_weight='balanced' | n_estimators=100, max_depth=None, min_samples_split=2 |
| MLP | scikit-learn 1.2.0 | hidden_layer_sizes=(128,64,32), max_iter=200, solver='adam', random_state=42 | activation='relu', alpha=0.0001 |

Abbreviations: LR, logistic regression; SVM, support vector machine; DT, decision tree, RF, random forest, XGBoost, extreme gradient boosting, MLP, multilayer perceptron.

**Table S6** Radiomics Quality Score (RQS) components and our study’s performance.

| **Criterion** | **Description** | **Our study** | **Score** |
| --- | --- | --- | --- |
| 1 | Image protocol quality: protocols well documented | Yes (Table S2) | +2 |
| 2 | Multiple segmentations and perturbing segmentations | Yes (Methods: Image perturbation) | +2 |
| 3 | Phantom study or analysis of feature robustness | Yes (Methods: Repeatability) | +2 |
| 4 | Imaging at multiple timepoints | Yes (Methods: Time serial prediction) | +2 |
| 5 | Feature reduction methods | Yes (LASSO, F-test, correlation filtering) | +2 |
| 6 | Multivariable analysis with non-radiomic features | No | 0 |
| 7 | Biological correlates | No | 0 |
| 8 | Cut-off analysis | No | 0 |
| 9 | Discrimination statistics with resampling | Yes (Methods: Statistical Analysis) | +2 |
| 10 | Calibration analysis with resampling | Yes (Methods: Statistical Analysis) | +2 |
| 11 | Prospective study | No | 0 |
| 12 | Validation: dataset from another institute | Yes (external test set) | +3 |
| 13 | Comparison to ‘gold standard’ | Yes (postoperative pathology) | +2 |
| 14 | Potential clinical utility | No | 0 |
| 15 | Cost-effectiveness analysis | No | 0 |
| 16 | Open science and data | Yes (code: GitHub, data available on request) | +2 |
| Total |  |  | 17 / 36 (47.22%) |

**Table S7.** Detailed parameter configuration for image perturbation using the MIRP toolkit.

| **Parameter** | **Description** | **Default value** |
| --- | --- | --- |
| perturbation_noise_level | Standard deviation of Gaussian noise (mean 0) | As present in the image (derived from image intensity variability) |
| perturbation_rotation_angles | Sub‑voxel translation distance as fraction of voxel spacing | A fraction of the voxel spacing (not fixed) |
| perturbation_translation_fraction | In‑plane rotation angle around the z‑axis | 0.5° |
