# Supplementary figures and images for: Moran's I-driven habitat radiomics: A biologically plausible and temporally robust approach for risk stratification of lung adenocarcinoma invasiveness
Source: Eur J Radiol Open. 2026 Jul 14;17:100792. doi: 10.1016/j.ejro.2026.100792 (PMC13377481; doi:10.1016/j.ejro.2026.100792)

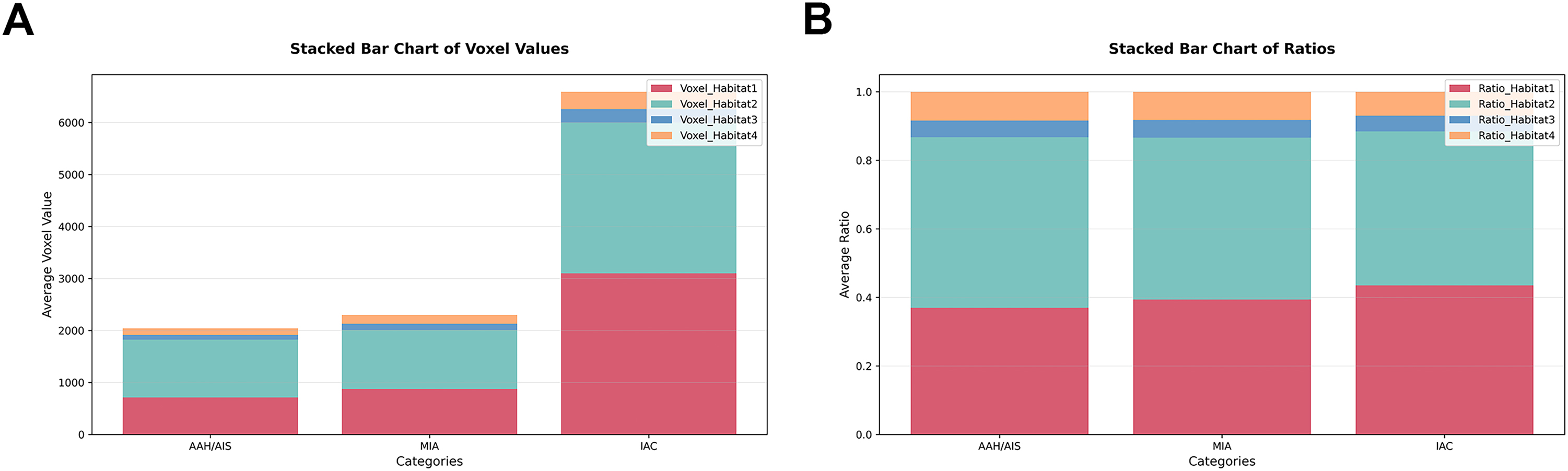

Supplement: Supplementary file 3 — Supplementary material [file mmc3.jpg]

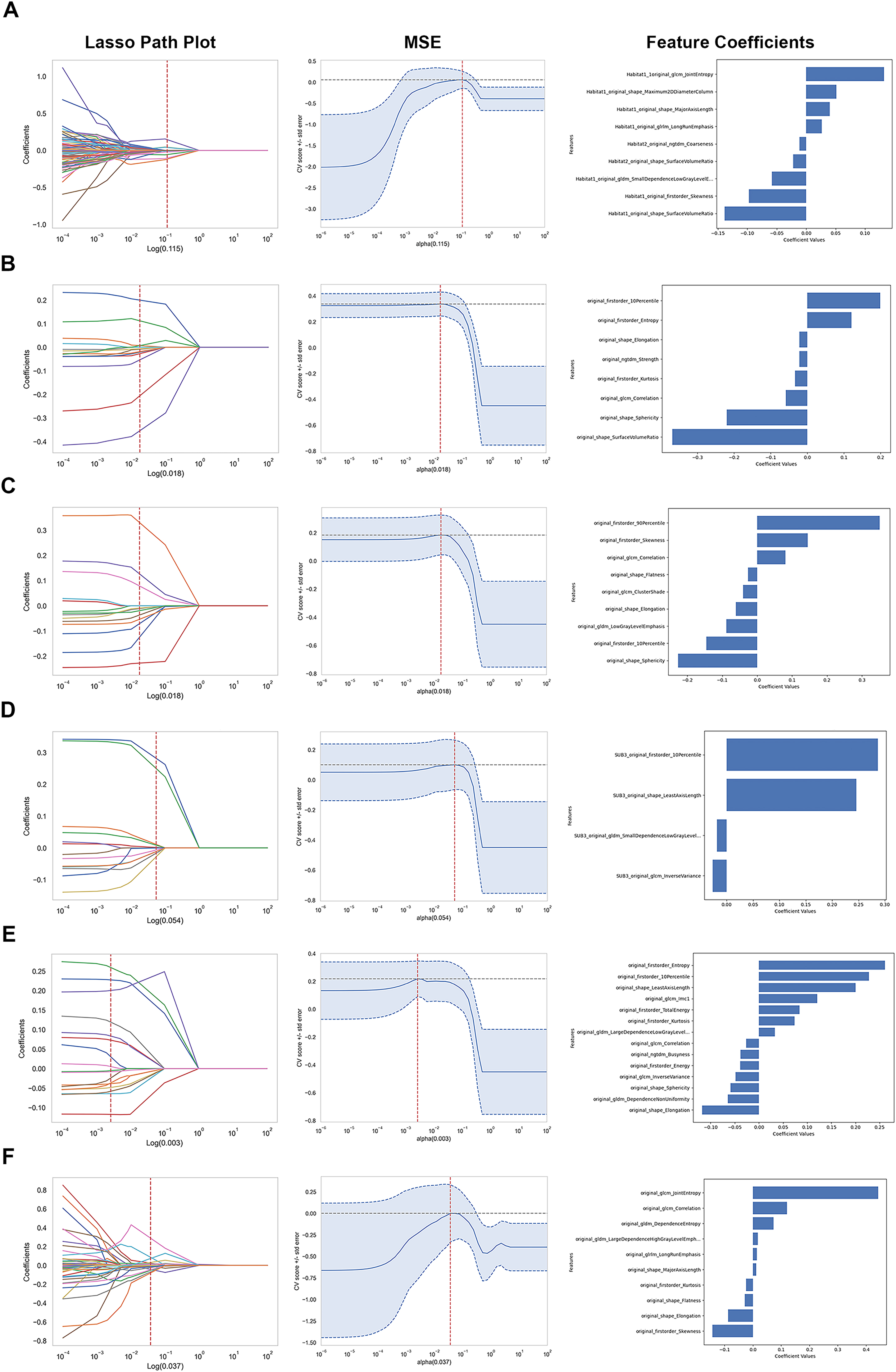

Supplement: Supplementary file 4 — Supplementary material [file mmc4.jpg]

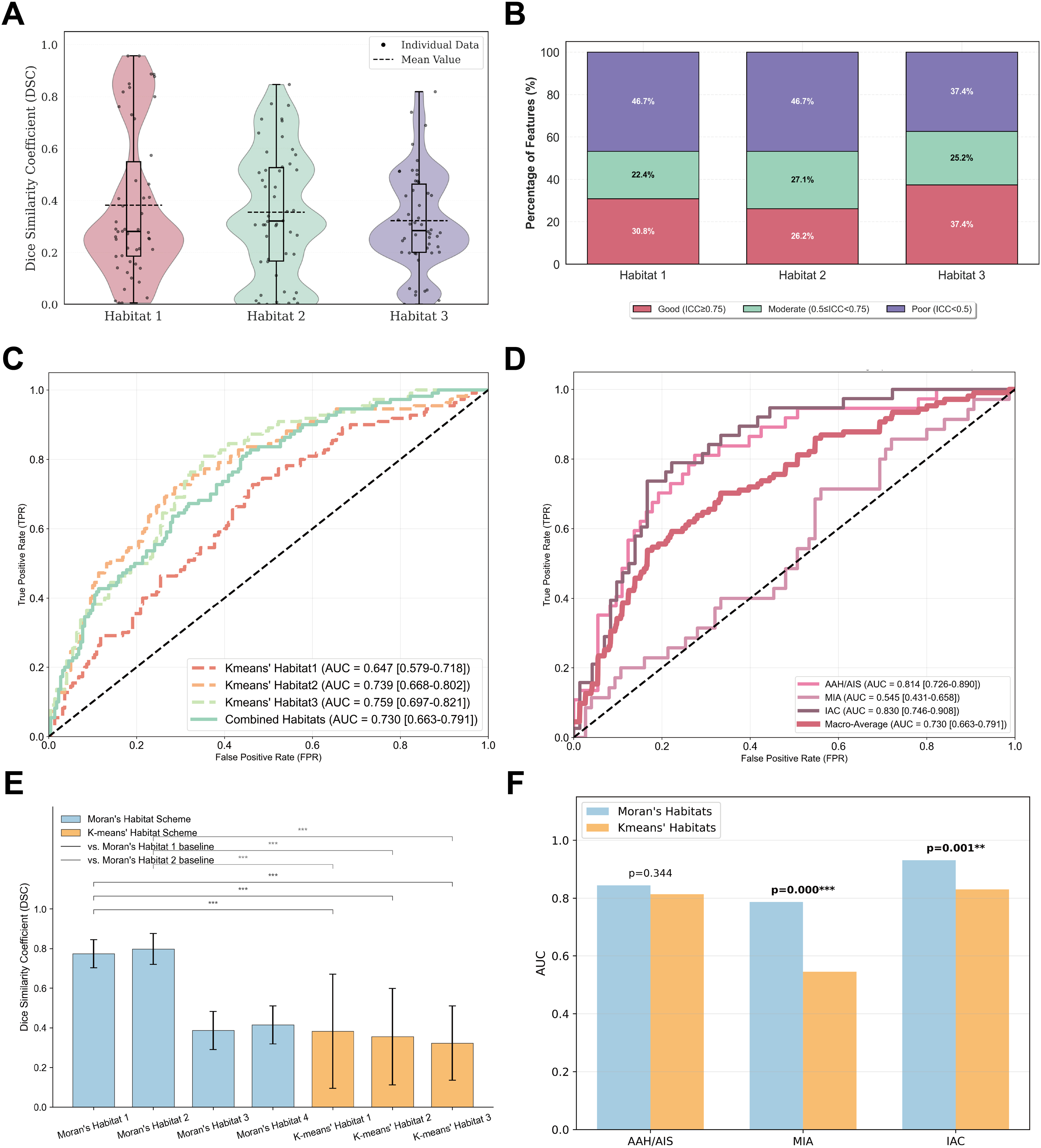

Supplement: Supplementary file 5 — Supplementary material [file mmc5.jpg]

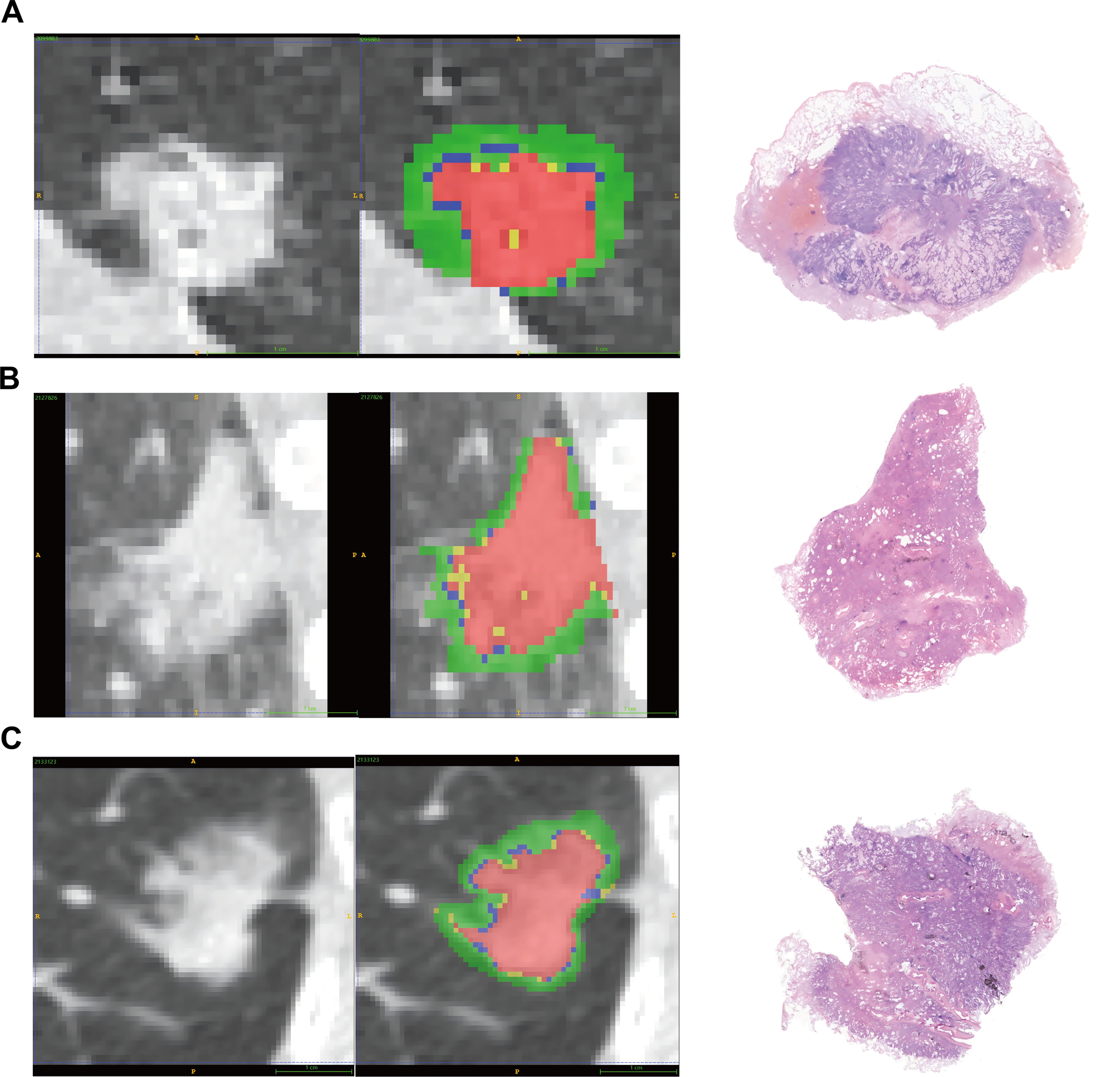

Supplement: Supplementary file 6 — Supplementary material [file mmc6.jpg]
